# Free, bioavailable 25-hydroxyvitamin D levels and their association with diabetic ketoacidosis in children with type 1 diabetes at diagnosis

**DOI:** 10.3389/fendo.2022.997631

**Published:** 2022-10-20

**Authors:** Yunsoo Choe, Yun Jeong Lee, Jae Hyun Kim, Kyunghoon Lee, Choong Ho Shin, Young Ah Lee, Junghan Song

**Affiliations:** ^1^ Department of Pediatrics, Seoul National University Children’s Hospital, Seoul National University College of Medicine, Seoul, South Korea; ^2^ Department of Pediatrics, Seoul National University Bundang Hospital, Seongnam, South Korea; ^3^ Department of Laboratory Medicine, Seoul National University Bundang Hospital, Seongnam, South Korea

**Keywords:** diabetes mellitus type 1, pediatrics, diabetic ketoacidosis (DKA), 25-hydroxyvitamin D (25OHD), vitamin D-binding protein

## Abstract

**Background:**

Considering the roles of 25-hydroxyvitamin D (25OHD) in glucose homeostasis and immune modulation, vitamin D deficiency may be related to the development of type 1 diabetes (T1DM) and diabetic ketoacidosis (DKA). We evaluated the total, free, bioavailable 25OHD levels and vitamin D binding protein (VDBP) levels and genotypes between T1DM patients and controls.

**Methods:**

This retrospective, cross-sectional study included 84 children with T1DM (38 boys and 46 girls, 8.0 ± 3.6 years) and 1:1 age- and sex-matched healthy controls. A multiplex liquid chromatography-tandem mass spectrometry-based assay was used to simultaneously measure vitamin D metabolites.

**Results:**

Patients with T1DM had lower levels of total 25OHD (16.3 ± 5.1 vs. 19.9 ± 6.5 ng/mL, *P*< 0.001) and VDBP (146.0 ± 27.8 vs. 224.9 ± 36.1 µg/mL, *P* = 0.001), but higher free 25OHD (8.0 ± 2.5 vs. 6.5 ± 2.3 pg/mL, *P*< 0.001) than controls. Patients who presented with DKA had lower levels of 25OHD in the total (15.0 ± 4.6 vs. 17.6 ± 5.2 ng/mL, *P* = 0.020), free (7.5 ± 2.6 vs. 8.4 ± 2.4 pg/mL, *P* = 0.059), and bioavailable (2.3 ± 0.9 vs. 2.8 ± 0.8 ng/mL, *P* = 0.014) forms than those without DKA at the T1DM diagnosis. The lower the total, free, and bioavailable 25OHD levels at diagnosis, the lower the pH and HCO3-. The proportions of the VDBP genotypes did not differ between the patients and controls.

**Conclusion:**

Patients with T1DM had higher levels of free 25OHD than healthy children, despite lower levels of total 25OHD. However, patients with DKA exhibited lower levels of bioavailable 25OHD than those without DKA at the T1DM diagnosis. The lower the concentrations of free and bioavailable 25OHD, the more severe the acidosis at the initial T1DM presentation.

## Introduction

Sufficient vitamin D status has been emphasized since the classical and non-classical effects of vitamin D on overall health were reported (1). Vitamin D deficiency is more commonly reported in patients with type 1 diabetes mellitus (T1DM) than in healthy controls (2–5), and adequate vitamin D intake during early life has a preventive effect on the risk of developing T1DM (6). Although the underlying mechanisms are unknown, the beneficial effects of vitamin D on immune modulation (7), insulin synthesis, insulin secretion (8), and insulin sensitivity (9) are possible explanations.

Vitamin D binds to vitamin D binding protein (VDBP, 85–90%) or albumin (10–15%); only a small proportion circulates free (< 1%). According to the “free hormone hypothesis,” the free or albumin-bound form of 25-hydroxyvitamin D (25OHD) plays a pivotal role, while the VDBP-bound form serves as a reservoir (10). Although the relationship between low concentrations of total 25OHD and the severity of diabetic ketoacidosis (DKA) has been reported in patients with T1DM (11), no investigation of free or bioavailable vitamin D at the time of T1DM diagnosis in association with DKA or in comparison with healthy controls has been conducted.

Lower (12) or similar (13–15) VDBP concentrations have been reported in established T1DM patients. The VDBP concentration varies according to the VDBP genotype, of which the distribution differs by race (16). Additionally, different physiological conditions and disease status can affect the VDBP concentration (17, 18). As the total 25OHD level depends on the serum VDBP concentration, the relationship between VDBP variants, vitamin D metabolites, and disease severity at the time of the T1DM diagnosis should be investigated.

We recently simultaneously measured several 25OHD metabolites and VDBP isoforms *via* multiplex liquid chromatography-tandem mass spectrometry (LC-MS/MS) (19). Herein, we compared the concentrations of total, free, and bioavailable 25OHD in association with VDBP in healthy controls and children newly diagnosed with T1DM. We investigated whether vitamin D metabolites are related to the presence of DKA or disease severity at the time of T1DM diagnosis. We also compared the proportions of the VDBP genotypes between the T1DM and control groups and analyzed whether the VDBP genotypes affect the occurrence and severity of DKA.

## Methods

### Subjects

Children (< 18 years of age) with blood samples at the time of the T1DM diagnosis at Seoul National University Children’s Hospital were included in this study. T1DM was defined as insulin-dependence and having one or more autoimmune markers, including autoantibodies to glutamic acid decarboxylase, islet cells, insulin, or islet antigen-2 (20). Islet antigen-2 autoantibody positivity and titers have been recorded in a limited number of patients since it became measurable in 2018. Among the 298 blood samples obtained from T1DM children between April 2008 and April 2021, 87 were taken at the time of T1DM diagnosis. Finally, 84 T1DM cases were included in this study, after excluding 3 patients for a lack of biochemical information about ketoacidosis at diagnosis (**Supplementary Figure 1**). We used previous clinical and biochemical data from healthy control children (13, 21), and age- and sex-matched children (n = 84) were included in this study. Case–control matches were performed using the matchControls function in the e1071 package in R software (The R Foundation for Statistical Computing, Vienna, Austria), which computes pairwise dissimilarities between observations using Gower’s coefficients (22).

### Physical examination

Clinical information on height, weight, and pubertal status were collected. Body mass index (BMI) was calculated as the body weight divided by the height squared. The BMI and weight-for-height percentiles were assigned based on the 2017 Korean National Growth Chart (23). For children aged > 2 years, overweight and obesity were defined as ≥ 85th and< 95th percentiles of the BMI for age and sex and ≥ 95th percentile of the BMI for age and sex, respectively. For those aged< 2 years old, overweight was defined as ≥ 95th percentile of weight-for-height for age and sex. Seasonality at the time of T1DM diagnosis was classified as spring or summer (March – August) and fall or winter (September – February).

### Biochemical assessments

The blood samples were centrifuged at 1500 × g for 10 min within 6 h of collection and stored at −70°C for analysis. 25-Hydroxyvitamin D2 (25OHD_2_), 25-hydroxyvitamin D3 (25OHD_3_), 24,25-dihydroxyvitamin D3 (24,25OH_2_D_3_), and the VDBP isoforms were simultaneously quantified *via* multiplex LC-MS/MS. In brief, samples pretreated with hexane and trypsin were analyzed with an ACQUITY ultra-performance liquid chromatography (UPLC) system (Waters, Milford, MA, USA) using an HSS T3 column (2.1 × 50 mm, 1.8 μm) and Xevo TQ-S mass analyzer (Waters). The coefficient of variation for the three quality control materials was ± 10% and ± 20% at the lower limit of quantification. Additional details can be found elsewhere (19).

Vitamin D sufficiency was defined when total 25OHD (sum of 25OHD_2_ and 25OHD_3_) was ≥20 ng/mL, and low vitamin D (either vitamin D deficiency or insufficiency) was defined when total 25OHD was<20 ng/mL (24). Bioavailable vitamin D was defined as the sum of free vitamin D and albumin-bound vitamin D (25). Bikle’s formula (26) was used to calculate free and bioavailable vitamin D using the concentrations of serum albumin, VDBP, and total 25OHD, and the binding affinities for albumin and VDBP. Kalb and KVDBP represent the affinity constants between 25OHD and albumin (6 × 10^5^ M^-1^), and between 25OHD and VDBP (7 × 10^8^ M^-1^), respectively. The vitamin D metabolite ratio (VMR) was calculated as the ratio of 24,25OH_2_D_3_ to total 25OHD (27). According to the newly established reference of free 25OHD by Zeng et al. (28), free vitamin D sufficiency was defined when free 25OHD was ≥5.67 pg/mL.


$$\text{Free }25\text{OHD }=\frac{\text{total }25\text{OHD}}{1+\left({\text{K}}_{\text{alb}}\;\times \text{ albumin}\right)+\left({\text{K}}_{\text{VDBP}}\;\times \text{ VDBP}\right)}$$

Bioavailable 25 OHD


=free 25OHD+albumin−bound 25OHD   

$$=\text{free }25\text{OHD }\left(1+{\text{K}}_{\text{alb}}\;\times \text{ albumin}\right)$$

Vitamin D metabolite ratio (VMR, %)                                                      

$$=\;\frac{24,25{\text{OH}}_{2}{\text{D}}_{3}}{25{\text{OHD}}_{3}}\;\times \;100\;\;\;$$

The relative concentrations of free 25OHD to total 25OHD (free to total 25OHD ratio) and the relative concentrations of bioavailable 25OHD to total 25OHD (bioavailable to total 25OHD ratio) were expressed as percentages after matching the units.

Free to total 25 OHD ratio 


$$=\;\frac{\text{Free }25\text{OHD }\left(\text{pg}/\text{mL}\right)\text{ x }10^{-3}}{\text{Total }25\text{OHD }\left(\text{ng}/\text{mL}\right)}\;\times \;100$$

Bioavailable to total 25 OHD ratio (%)


$$=\;\frac{\text{Bioavailable }25\text{OHD }\left(\text{ng}/\text{mL}\right)}{\text{Total }25\text{OHD }\left(\text{ng}/\text{mL}\right)}\;\times \;100$$

Serum concentrations of calcium, phosphorus, albumin, and creatinine were collected. The estimated glomerular filtration rate (eGFR) was calculated using the bedside Schwartz Estimate Equation [eGFR = 0.413 × height (cm)/serum creatinine (mg/dL)] (29). Laboratory results for glycated hemoglobin (HbA1c), pH, bicarbonate (HCO3-), serum C-peptide, and the positivity status and titers of diabetes-associated autoantibodies were collected for the T1DM cases. HbA1c was measured using the National Glycohemoglobin Standardization Program certified method. DKA was defined when the pH of the venous blood was< 7.3 and ketonuria or ketonemia was confirmed when the serum glucose level was > 200 mg/dL. DKA severity was classified as severe (pH< 7.1 or HCO3-< 5 mmol/L), moderate (7.1 ≤ pH< 7.2 or 5 mmol/L ≤ HCO3-< 10 mmol/L), or mild (7.2 ≤ pH< 7.3 or 10 mmol/L ≤ HCO3-< 15 mmol/L).

### Statistical analysis

All statistical analyses were performed using R version 4.0.5 software. All continuous variables are expressed as median [25th–75th percentiles] or mean ± standard deviation. The Shapiro-Wilk test was used to assess normality. Variables with a skewed distribution were natural log-transformed. Student’s t-test or the Mann-Whitney U test was used to compare continuous variables, and the chi-square test or Fisher’s exact test was used to compare categorical variables between the two groups. Comparisons among three groups were conducted with the Kruskal-Wallis test for non-parametric variables, and one-way analysis of variance (ANOVA) or Welch’s ANOVA for parametric variables with homogeneous or heterogeneous variances, respectively. Bonferroni’s method was used for the posthoc analysis. Spearman’s correlation analysis was conducted to evaluate the relationships between vitamin D metabolites and clinical and biochemical parameters. *P*-values< 0.05 were considered significant.

## Results

### Clinical and biochemical characteristics of the T1DM cases and healthy controls

The clinical and biochemical data of the T1DM cases and healthy controls are described in **Table 1**. Age, sex, pubertal status, the proportion of overweight or obesity, and seasonality did not differ between the two groups. Total, free, and bioavailable 25OHD levels and VDBP concentrations did not differ according to normal weight and overweight or obesity status within the T1DM cases and healthy controls, respectively. T1DM patients had significantly lower levels of albumin (3.5 ± 0.5 vs. 4.5 ± 0.4 g/dL, *P*< 0.001) and eGFR (82.9 ± 20.9 vs. 99.5 ± 15.0 mL/min/1.73 m^2^, *P*< 0.001) than controls. The total 25OHD levels were significantly lower (16.3 ± 5.1 vs. 19.9 ± 6.5 ng/mL, *P*< 0.001) with a lower proportion of vitamin D sufficiency (20.2 vs. 46.4%, *P* = 0.001) in children with T1DM than the controls. Children with T1DM had significantly lower concentrations of VDBP (146.0 ± 27.8 vs. 224.9 ± 36.1 µg/mL, *P*< 0.001) but higher levels of free 25OHD (8.0 ± 2.5 vs. 6.5 ± 2.3 pg/mL, *P*< 0.001) with a higher frequency of free vitamin D sufficiency (83.3 vs. 59.5%, *P* = 0.001) than the controls, although bioavailable 25OHD levels did not differ between the two groups. The free to total 25OHD ratio (0.050 ± 0.009 vs. 0.033 ± 0.005%) and bioavailable to total 25OHD ratio (15.5 ± 2.9 vs. 13.2 ± 1.8%) were significantly higher in the T1DM group than the controls, respectively (*P*< 0.001 for both). The concentration of 24,25OH_2_D_3_ was lower in T1DM patients (0.9 ± 0.5 vs. 1.1 ± 0.6 ng/mL, *P* = 0.006), but the VMR was similar between the two groups (**Table 1**).

**Table 1:** Demographic characteristics and biochemical parameters between healthy control children and patients with type 1 diabetes, grouped by presence of diabetic ketoacidosis at diagnosis.

	Control (n = 84)	T1DM total (n = 84)	DKA (-) T1DM (n = 43)	DKA (+) T1DM (n = 41)
*Demographic characteristics*				
Age, year	9.2 ± 2.2	8.0 ± 3.6	8.1 ± 3.2	7.9 ± 4.1
Boys, n (%)	38 (45.2)	38 (45.2)	21 (48.8)	17 (41.5)
Prepuberty, n (%)	63 (75.0)	55 (65.5)	30 (69.8)	25 (61.0)
Overweight or obesity, n (%)	9 (10.7)	8 (9.5)	5 (11.6)	3 (7.3)
Spring or summer: fall or winter, n (%)	44: 40 (52.4: 47.6)	48: 36 (57.1: 42.9)	25: 18 (58.1: 41.9)	23: 18 (56.1: 43.9)
*Biochemical parameters*				
Calcium, mg/dL	9.6 ± 0.4	9.7 ± 0.7	9.8 ± 0.5	9.6 ± 0.9
Phosphorus, mg/dL	5.2 ± 0.5	4.1 ± 0.8	4.5 ± 0.6^a^	3.7 ± 0.9^a,b^
Albumin, g/dL	4.5 ± 0.4	3.5 ± 0.5	3.6 ± 0.4^a^	3.4 ± 0.6^a^
eGFR, mL/min/1.73 m^2^	99.5 ± 15.0	82.9 ± 20.9	88.4 ± 15.8^a^	77.3 ± 24.1^a,b^
*Vitamin D metabolites*				
Vitamin D sufficiency^c^, n (%)	39 (46.4)	17 (20.2)	11 (25.6)	6 (14.6)^a^
Total 25OHD, ng/mL	19.9 ± 6.5	16.3 ± 5.1	17.6 ± 5.2	15.0 ± 4.6^a^
25-hydroxyvitamin D3, ng/mL	19.5 ± 6.6	16.0 ± 5.1	17.3 ± 5.2	14.7 ± 4.6^a^
25-hydroxyvitamin D2, ng/mL	1.1 ± 1.4	0.3 ± 0.2	0.3 ± 0.2^a^	0.2 ± 0.1^a^
Vitamin D binding protein, μg/mL	224.9 ± 36.1	146.0 ± 27.8	147.7 ± 26.7^a^	144.2 ± 29.1^a^
Free vitamin D sufficiency^d^, n (%)	50 (59.5)	70 (83.3)	40 (93.0)^a^	30 (73.2)
Free 25OHD, pg/mL	6.5 ± 2.3	8.0 ± 2.5	8.4 ± 2.4^a^	7.5 ± 2.6
Bioavailable 25OHD, ng/mL	2.6 ± 0.9	2.5 ± 0.9	2.8 ± 0.8	2.3 ± 0.9^b^
Free to total 25OHD ratio, %	0.033 ± 0.005	0.050 ± 0.009	0.049 ± 0.009^a^	0.051 ± 0.010^a^
Bioavailable to total 25OHD ratio, %	13.2 ± 1.8	15.5 ± 2.9	15.8 ± 2.6^a^	15.3 ± 3.2^a^
24,25-dihydroxyvitamin D3, ng/mL	1.1 ± 0.6	0.9 ± 0.5	1.1 ± 0.6	0.7 ± 0.4^a,b^
Vitamin D metabolite ratio, %	5.6 ± 1.5	5.3 ± 2.0	6.0 ± 2.1	4.6 ± 1.6^a,b^
*Biochemical markers related to diabetes*				
DKA severity (mild: moderate: severe)	–	–	–	24: 7: 10 (58.5: 17.1: 24.4)
pH	–	–	7.4 ± 0.0	7.2 ± 0.1
HCO3, mmol/L	–	–	23.1 ± 4.5	12.4 ± 6.0
Initial HbA1c, %	–	–	12.0 ± 2.3	12.5 ± 1.7
Serum C-peptide, ng/mL	–	–	0.7 ± 0.7	0.4 ± 0.4
Autoantibodies positivity	–	–		
GAD autoantibody (+)	–	–	40 (95.2)	33 (80.5)
islet cell autoantibody (+)	–	–	3 (7.0)	7 (17.1)
insulin autoantibody (+)	–	–	12 (27.9)	22 (55.0)
IA-2 autoantibody (+)	–	–	5 (62.5)	7 (100.0)
Number of autoantibody (+), 1:2:3:4	–	–	29: 11: 3: 0 (67.4: 25.6: 7.0: 0.0)	18: 19: 3: 1 (43.9: 46.3: 7.3: 2.4)

Values are presented as mean ± standard deviation or number (%).

T1DM, type 1 diabetes mellitus; DKA, diabetic ketoacidosis; eGFR, estimated glomerular filtration rate; 25OHD, 25-hydroxyvitamin D.

Missing values for initial glucose (n = 1), GAD autoantibody (n = 1), Insulin autoantibody (n = 1), IA-2 autoantibody (n = 69).

a P< 0.02 differs from control after Bonferroni correction.

b P< 0.02 differs from DKA (-) T1DM after Bonferroni correction.

c Vitamin D sufficiency was defined when total 25OHD ≥ 20 ng/mL.

d Free vitamin D sufficiency was defined when free 25OHD ≥ 5.67 pg/mL.

### Comparisons of vitamin D metabolites according to whether children with T1DM presented with DKA at the time of diagnosis

Next, vitamin D metabolites in blood samples taken at the time of the T1DM diagnosis were compared according to whether the T1DM patients presented with DKA [DKA (+) T1DM, n = 41] or not [DKA (–) T1DM, n = 43] at diagnosis. The DKA severity at diagnosis was classified as mild (n = 24, 58.5%), moderate (n = 7, 17.1%), or severe (n = 10, 24.4%) (**Table 1**). Although the initial HbA1c levels did not differ between the two groups, the serum C-peptide levels were lower in the DKA (+) than in the DKA (–) group (*P* = 0.027). Although the number of positive autoantibodies did not differ between the two groups, the DKA (+) group had a higher proportion of insulin autoantibody positivity compared to the DKA (–) group (55.0 vs. 27.9%, *P* = 0.022).

**Figure 1:**
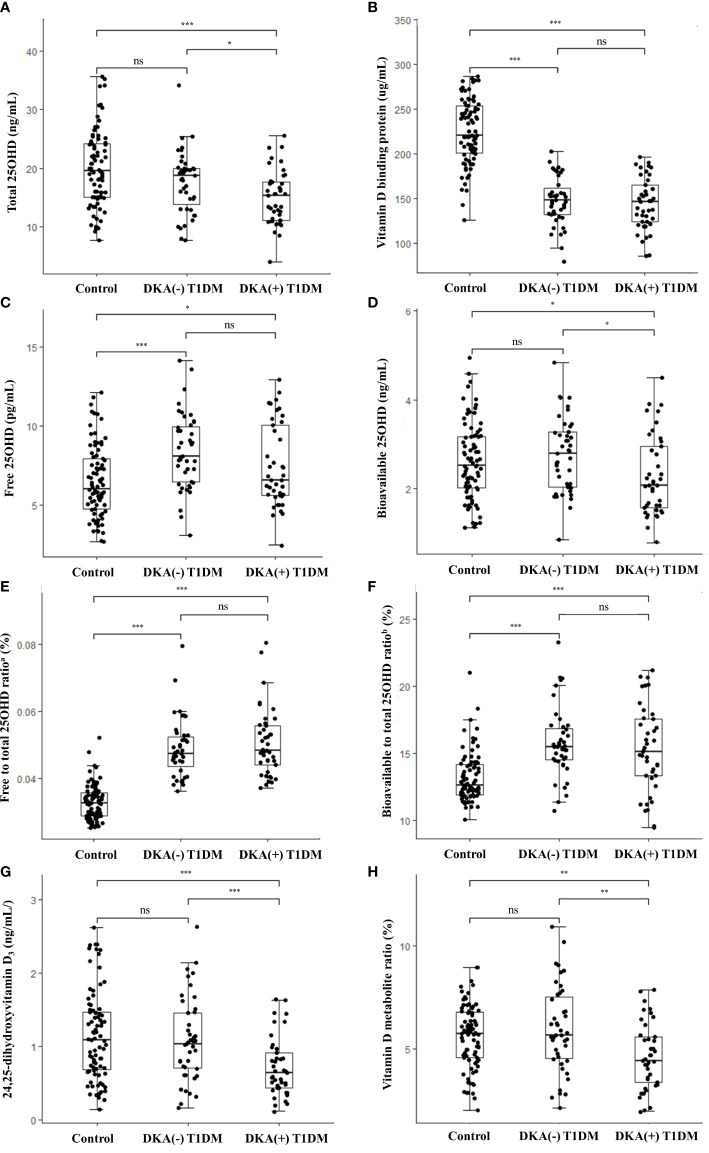
Vitamin D metabolite levels in the study population. Box-and-whisker plots of the concentrations of **(A)** total 25OHD, **(B)** vitamin D binding protein, **(C)** free 25OHD, **(D)** bioavailable 25OHD, **(E)** free to total 25OHD ratio, **(F)** bioavailable to total 25OHD ratio, **(G)** 24,25-dihydroxyvitamin D_3_, and **(H)** vitamin D metabolite ratio. Comparisons among three groups were conducted with Kruskal-wallis test for non-parametric and one-way analysis of variance (ANOVA) or Welch’s ANOVA for parametric variables with homogeneous or heterogeneous variances. The thick lines in the boxes indicate the medians and the whiskers indicate the maximum and minimum values, respectively. Statistically significant differences are indicated by asterisks (*P< 0.05, **P< 0.01, ***P< 0.001). T1DM, type 1 diabetes mellitus; DKA, diabetes ketoacidosis; 25OHD, 25-hydroxyvitamin D; ns, non-significant. ^a^Free to total 25OHD ratio (%) = [Free 25OHD (pg/mL) x 10^-3^]/[Total 25OHD (ng/mL)] x 100 ^b^Bioavailable to total 25OHD ratio (%) = [Bioavailable 25OHD (ng/mL)]/[Total 25OHD (ng/mL)] x 100.

**Figure 1** shows a comparison of the vitamin D metabolites among the three groups [healthy controls, DKA (–) T1DM, and DKA (+) T1DM groups]. The DKA (+) group had the lowest levels of total 25OHD compared to the control (15.0 ± 4.6 vs. 19.9 ± 6.5 ng/mL, *P*< 0.001) and DKA (–) (15.0 ± 4.6 vs. 17.6 ± 5.2 ng/mL, *P* = 0.020) groups (**Figure 1A**). The serum concentrations of VDBP in the DKA (–) and DKA (+) groups were significantly lower than those in the control group (147.7 ± 26.7 in the DKA (–) and 144.2 ± 29.1 in the DKA (+) vs. 224.9 ± 36.1 µg/mL in the control group, *P*< 0.001 for both) without a difference between the DKA (+) and DKA (–) groups (**Figure 1B**). The free and bioavailable 25OHD levels were highest in the DKA (–) group among the three groups. The free 25OHD levels increased significantly in the DKA (–) and DKA (+) groups compared to the control group (8.4 ± 2.4 in DKA (–) and 7.5 ± 2.6 in DKA (+) vs. 6.5 ± 2.3 pg/mL in the control group, *P*< 0.001 and *P* = 0.047, respectively). Bioavailable 25OHD was lower in the DKA (+) than the DKA (–) group (2.3 ± 0.9 vs. 2.8 ± 0.8 ng/mL, *P* = 0.014), with no difference between the control and DKA (–) groups (**Figures 1C, D**). The free to total 25OHD ratio and the bioavailable to total 25OHD ratio were significantly higher in the DKA (–) and DKA (+) groups than in the controls (*P*< 0.001 for both), without differences between the DKA (–) and DKA (+) groups (**Figures 1E, F**). The concentrations of 24,25OH_2_D_3_ and VMR were significantly lower in the DKA (+) group than in the control and DKA (–) groups (24,25OH_2_D_3_: 0.7 ± 0.4 in the DKA (+) vs. 1.1 ± 0.6 in the control and 1.1 ± 0.6 ng/mL in the DKA (–) group, *P*< 0.001 and *P* = 0.001, respectively) (VMR: 4.6 ± 1.6 in the DKA (+) vs. 5.6 ± 1.5 in the control and 6.0 ± 2.1% in the DKA (–) group, *P* = 0.002 and *P* = 0.003, respectively) (**Figures 1G, H** and **Table 1**).

### Vitamin D metabolites and the biochemical markers in T1DM patients

**Table 2** shows the association between vitamin D metabolites and biochemical parameters reflecting disease severity at the time of the T1DM diagnosis. Total, free, and bioavailable 25OHD levels were positively correlated with pH (*P* = 0.011 for total 25OHD, *P* = 0.027 for free 25OHD, and *P* = 0.006 for bioavailable 25OHD) and HCO3- (*P* = 0.033 for total 25OHD, *P* = 0.037 for free 25OHD, and *P* = 0.031 for bioavailable 25OHD). The serum VDBP concentration was inversely associated with the initial HbA1c value (*P* = 0.012).

**Table 2:** Association of vitamin D metabolites with biochemical parameters at the time of T1DM diagnosis.

	Total 25OHD^a^, ng/mL	Free 25OHD^a^, pg/mL	Bioavailable 25OHD^a^, ng/mL	Vitamin D binding protein, μg/mL
Initial HbA1c, %	-0.086	0.067	-0.017	-0.270^b^
pH^a^	0.282^b^	0.245^b^	0.303^b^	0.079
HCO3^a^, mmol/L	0.240^b^	0.203^b^	0.242^b^	0.067

Spearman’s correlation coefficients are shown for paired variables of interest.

T1DM, type 1 diabetes mellitus; 25OHD, 25-hydroxyvitamin D.

a Log-transformed form.

b Statistical significance at P<0.05.

### Distribution of VDBP isoforms and diabetes biochemical parameters according to VDBP genotype

When the VDBP genotypes were analyzed, Gc1f/2 and Gc1s/1f were equally detected in 21.8% (n = 19 each) of the T1DM patients, and Gc1f/1f was observed in 33.3% (n = 29) of the healthy children (**Supplementary Figure 2**). No significant differences in the proportions of the VDBP genotypes were found between the T1DM and control groups. The presence of DKA and the biochemical parameters reflecting disease severity (initial HbA1c, pH, and HCO3-) at the time of T1DM diagnosis were not significantly different according to the VDBP genotype (**Supplementary Table 1**).

## Discussion

In this study, pediatric patients with T1DM had lower total 25OHD levels but higher free 25OHD levels than healthy children. The ratios of free and bioavailable 25OHD to total 25OHD significantly increased, and the VDBP levels predominantly decreased in patients with T1DM compared to the healthy control group. When the T1DM patients were compared according to the presence of DKA at the initial presentation, the DKA (+) group had lower levels of bioavailable 25OHD, 24,25OH_2_D_3_, and VMR than the DKA (–) group. The total, free, and bioavailable 25OHD levels were significantly associated with DKA severity at diagnosis. The distribution of the VDBP genotypes was not different according to the T1DM diagnosis or the severity of acidosis.

The total 25OHD level was lower, but the free 25OHD level was higher, in the T1DM patients than in the healthy controls. Previous studies have suggested that vitamin D deficiency contributes to the development of T1DM (2–6), an autoimmune disease characterized by absolute insulin insufficiency. Vitamin D has immunomodulatory effects by reducing pro-inflammatory cytokines in immune cells through binding to vitamin D receptor (VDR) (7, 30). Additionally, vitamin D increases insulin synthesis and secretion by inducing the expression of genes involved in insulin secretion by pancreatic β cells (8, 31, 32) and increases insulin sensitivity by stimulating the expression of insulin receptors in skeletal muscle and adipocytes (9, 32, 33). Serum levels of total 25OHD have traditionally been used as the standard marker of vitamin D status (34). However, the “free hormone hypothesis” proposes that most cells, except those expressing the megalin-cubilin complex, such as renal cells, take up the non-protein-bound form of 25OHD (10, 35), suggesting that free or bioavailable 25OHD is a more accurate indicator of vitamin D status than total 25OHD (35). Considering the role of 25OHD in glucose homeostasis and this “free hormone hypothesis,” increasing the levels of free 25OHD in patients with T1DM may be a compensatory action for insulin deficiency.

In this study, T1DM patients had significantly lower VDBP and albumin levels, and higher ratios of free and bioavailable 25OHD to total 25OHD than controls. Their decreased protein levels may have resulted from decreased production (36) or increased excretion (14). Jain et al. (36) reported that plasma levels of VDBP significantly decrease when human monocytes, one of the organs that produce VDBP, are exposed to high levels of glucose. In addition, Thrailkill et al. (14) reported that VDBP is excessively filtered in the renal tubules before the development of microalbuminuria in patients with T1DM. Although we could not quantify urinary albumin or VDBP levels due to a lack of urine taken at the time of diagnosis, we speculate that decreased protein production (36) or increased protein excretion (14) may decrease VDBP and albumin levels in diabetic patients, contributing to increased free or bioavailable 25OHD levels in T1DM cases.

The levels of bioavailable 25OHD, 24,25OH_2_D_3_, VMR, and eGFR were significantly lower in the DKA (+) T1DM group than in the DKA (–) T1DM group, with inverse relationships of free and bioavailable 25OHD levels with the severity of acidosis. The mechanism underlying their inverse relationship remains to be determined. Considering the role of vitamin D in glucose homeostasis, reduced levels of free and bioavailable 25OHD in association with the presence of DKA and the severity of acidosis may represent failure to maintain glucose homeostasis. In addition, 25OHD_3_ is not converted efficiently to its active form of 1,25-dihydroxyvitamin D3 (1,25OH_2_D_3_) in patients with impaired renal function (37). Although we could not measure active 1,25OH_2_D_3_ levels in this study, the low eGFR in the DKA (+) T1DM group may have adversely affected the conversion of 25OHD_3_ into the 1,25OH_2_D_3_ active form. Furthermore, the concentration of 24,25OH_2_D_3_ and the VMR reflect VDR activity, as increased binding and activation of the VDR in response to 1,25OH_2_D_3_ also induces conversion of 25OHD into 24,25OH_2_D_3_ (38). As mentioned, vitamin D stimulates insulin production by upregulating insulin gene transcription and binding to the VDR (8, 32). Hence, reduced VDR activity represented by decreased levels of 24,25OH_2_D_3_ and VMR in the DKA (+) T1DM group cannot render active vitamin D more effective. Taken together, these results suggest that reduced levels of free and bioavailable vitamin D, as well as possible insufficient conversion to active vitamin D in cases with impaired renal function, and reduced VDR activity may interact with the presence of DKA in T1DM.

The VDBP allele frequency in our study subjects was similar to previously reported Korean genotyping results (19), and no significant differences were observed between T1DM and controls. A recent meta-analysis reported no significant association between VDBP polymorphisms and T1DM risk (39). Because all of our subjects were the same race and the sample size was too small, we could not analyze the role of VDBP polymorphisms in the development of T1DM or DKA.

The limitations of our study include its retrospective, cross-sectional design, which hindered assessing the causal relationship between free 25OHD and the pathogenesis of T1DM. Second, the sample size was relatively small. Third, we could not measure 1,25OH_2_D_3_, fibroblast growth factor 23, and intact parathyroid hormone levels due to a lack of blood taken at the time of the T1DM diagnosis. Fourth, the urinary excretion of VDBP could not be evaluated because we could not obtain adequate amount of urine sample at the time of diagnosis. However, this study had several strengths. We investigated various vitamin D metabolites simultaneously using LC-MS/MS in T1DM patients for the first time. As T1DM patients with various degrees of DKA were included, we assessed the relationship between vitamin D metabolites and the severity of acidosis.

In conclusion, T1DM patients exhibited significantly lower total 25OHD but higher free 25OHD levels than the healthy control group. The concentration of bioavailable 25OHD and the VDR activity markers were lower in patients presenting with DKA compared to those without DKA at the time of the T1DM diagnosis. It remains to be determined whether adequate levels of free and bioavailable 25OHD can help prevent the development of T1DM or DKA.

## Data availability statement

The original contributions presented in the study are included in the article/**Supplementary Material**. Further inquiries can be directed to the corresponding authors.

## Ethics statement

The studies involving human participants were reviewed and approved by institutional review board of Seoul National University Hospital (approval No. H-2105-162-1221). Written informed consent to participate in this study was provided by the participants’ legal guardian/next of kin.

## Author contributions

YC, CHS, YAL, and JS contributed to conception and design of the study. KL and JS analyzed samples. YC, JHK, CHS, YAL, and JS interpreted the data. YC wrote the first draft of the manuscript. YC, YJL, JHK, KL, CHS, YAL, and JS critically reviewed and edited the manuscript. All authors contributed to the article and approved the submitted version.

## Funding

This work was supported by the National Research Foundation of Korea (NRF) grant funded by the Korea government (Ministry of Science and ICT) (No. NRF 2019R 1F 1A1058922). - Recipient: JS - Website: https://www.nrf.re.kr/eng/index. This study was supported by the Seoul National University Hospital Research Fund (No. 04-2019-3060) - Recipient: CHS - Website: https://en.snu.ac.kr/research/units/institutes. The funders had no role in study design, data collection and analysis, decision to publish, or preparation of the manuscript.

## Acknowledgments

The authors would like to thank Sun-Hee Jun and Jeong Seon Lee for sample preparation and analysis.

## Conflict of interest

The authors declare that the research was conducted in the absence of any commercial or financial relationships that could be construed as a potential conflict of interest.

## Publisher’s note

All claims expressed in this article are solely those of the authors and do not necessarily represent those of their affiliated organizations, or those of the publisher, the editors and the reviewers. Any product that may be evaluated in this article, or claim that may be made by its manufacturer, is not guaranteed or endorsed by the publisher.
