# Supplementary material for: Free, bioavailable 25-hydroxyvitamin D levels and their association with diabetic ketoacidosis in children with type 1 diabetes at diagnosis
Source: Front Endocrinol (Lausanne). 2022 Oct 20;13:997631. doi: 10.3389/fendo.2022.997631 (PMC9631212; doi:10.3389/fendo.2022.997631)
Supplement: Supplementary file 1 [file DataSheet_1.docx]

**Supplementary Materials**

Supplementary Table 1. Biochemical parameters of diabetes at diagnosis according to VDBP polymorphism in type 1 diabetes children (n = 84)

Supplementary Figure Legends

Supplementary Table 1. Biochemical parameters of diabetes at diagnosis according to *VDBP* polymorphism in type 1 diabetes children (n = 84)

| *VDBP* Genotype | Gc2/2 (n = 11) | Gc1f/1f (n = 18) | Gc1f/2 (n = 19) | Gc1s/1s (n = 9) | Gc1s/2 (n = 8) | Gc1s/1f (n = 19) | *P* value^a^ |
| --- | --- | --- | --- | --- | --- | --- | --- |
| Presence of DKA | 7 (63.6) | 12 (66.7) | 9 (47.4) | 4 (44.4) | 3 (37.5) | 6 (31.6) | 0.316 |
| Initial HbA1c, % | 12.9  [11.2, 13.8] | 12.1  [11.4, 13.4] | 12.3  [10.3, 13.6] | 11.2  [9.8, 12.4] | 12.2  [11.2, 14.1] | 11.9  [10.8, 12.8] | 0.526 |
| pH | 7.3  [7.2, 7.4] | 7.3  [7.1, 7.3] | 7.3  [7.2, 7.4] | 7.3  [7.1, 7.4] | 7.3  [7.3, 7.4] | 7.4  [7.3, 7.4] | 0.266 |
| HCO3, mmol/L | 15.9  [8.7, 23.5] | 16.9  [12.1, 21.6] | 20.1  [12.9, 24.5] | 19.3  [9.9, 23.1] | 18.5  [13.6, 22.6] | 20.4  [14.3, 26.0] | 0.807 |

Data are expressed as median [25^th^-75^th^ percentiles] or number (%)

VDBP, vitamin D binding protein; DKA, diabetic ketoacidosis

^a^*P* value by Kruskal-Wallis test or Fisher’s exact test.

**Supplementary Figure legends**

Supplementary Fig. 1. Flow chart of the study population. T1DM, type 1 diabetes mellitus

Supplementary Fig. 2. Distribution of *VDBP* genotypes in type 1 diabetes mellitus patients and healthy control. T1DM, type 1 diabetes mellitus
