# Supplementary figures and images for: Free, bioavailable 25-hydroxyvitamin D levels and their association with diabetic ketoacidosis in children with type 1 diabetes at diagnosis
Source: Front Endocrinol (Lausanne). 2022 Oct 20;13:997631. doi: 10.3389/fendo.2022.997631 (PMC9631212; doi:10.3389/fendo.2022.997631)

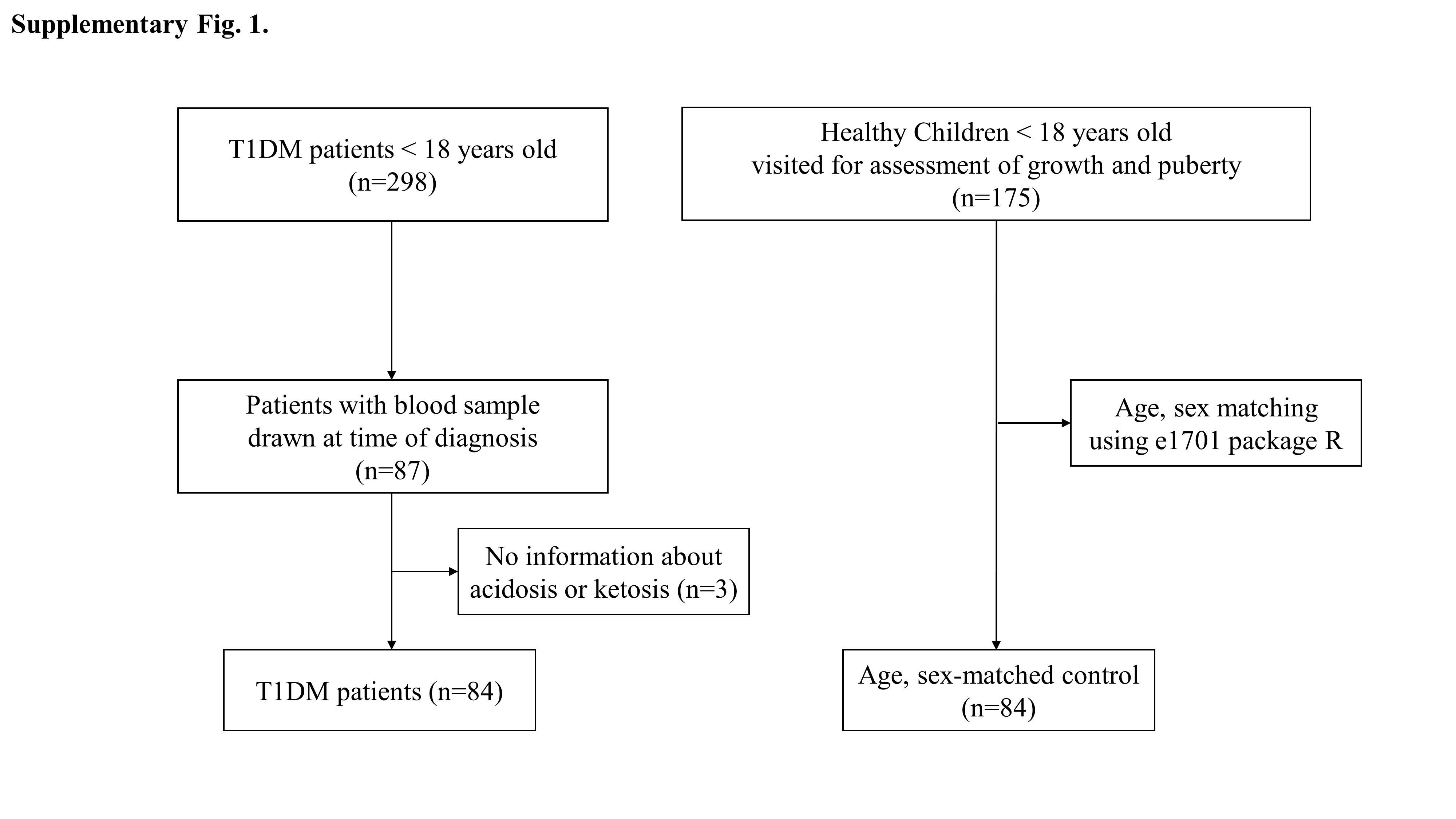

Supplement: Supplementary file 2 [file Image_1.tif]

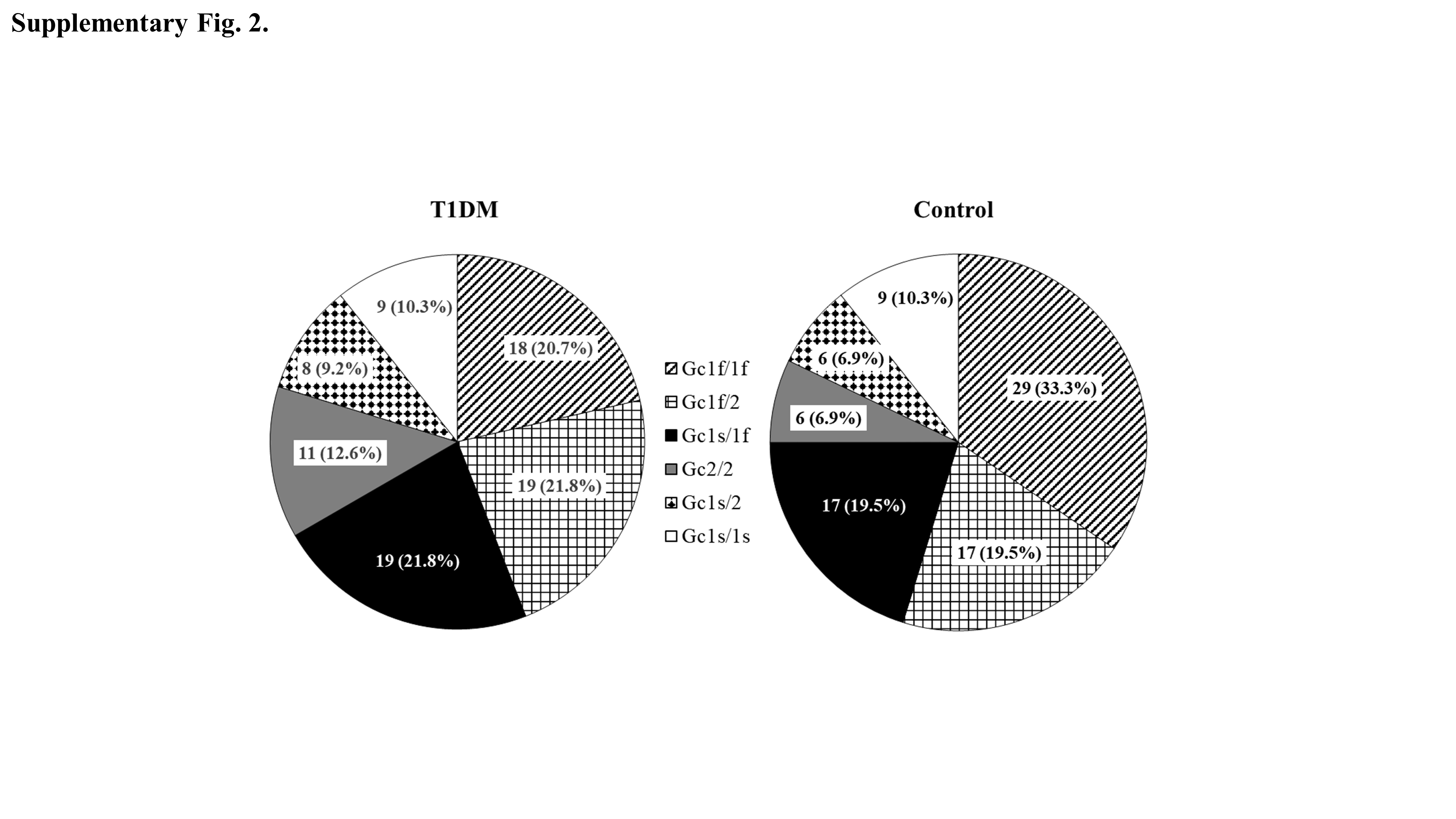

Supplement: Supplementary file 3 [file Image_2.tif]
